# Amyloid-β oligomerization monitored by single-molecule stepwise photobleaching

**DOI:** 10.1016/j.ymeth.2020.06.007

**Published:** 2021-09

**Authors:** Lara Dresser, Patrick Hunter, Fatima Yendybayeva, Alex L. Hargreaves, Jamieson A.L. Howard, Gareth J.O. Evans, Mark C. Leake, Steven D. Quinn

**Affiliations:** a Department of Physics, University of York, Heslington YO10 5DD, UK; b Department of Biology, University of York, Heslington YO10 5DD, UK; c York Biomedical Research Institute, University of York, Heslington YO10 5DD, UK

**Keywords:** Single-molecule TIRF, Photobleaching, Amyloid oligomerization, Calcium sensing, Fluorescence

## Abstract

A major hallmark of Alzheimer’s disease is the misfolding and aggregation of the amyloid- β peptide (Aβ). While early research pointed towards large fibrillar- and plaque-like aggregates as being the most toxic species, recent evidence now implicates small soluble Aβ oligomers as being orders of magnitude more harmful. Techniques capable of characterizing oligomer stoichiometry and assembly are thus critical for a deeper understanding of the earliest stages of neurodegeneration and for rationally testing next-generation oligomer inhibitors. While the fluorescence response of extrinsic fluorescent probes such as Thioflavin-T have become workhorse tools for characterizing large Aβ aggregates in solution, it is widely accepted that these methods suffer from many important drawbacks, including an insensitivity to oligomeric species. Here, we integrate several biophysics techniques to gain new insight into oligomer formation at the single-molecule level. We showcase single-molecule stepwise photobleaching of fluorescent dye molecules as a powerful method to bypass many of the traditional limitations, and provide a step-by-step guide to implementing the technique *in vitro*. By collecting fluorescence emission from single Aβ(1–42) peptides labelled at the N-terminal position with HiLyte Fluor 555 via wide-field total internal reflection fluorescence (TIRF) imaging, we demonstrate how to characterize the number of peptides per single immobile oligomer and reveal heterogeneity within sample populations. Importantly, fluorescence emerging from Aβ oligomers cannot be easily investigated using diffraction-limited optical microscopy tools. To assay oligomer activity, we also demonstrate the implementation of another biophysical method involving the ratiometric imaging of Fura-2-AM loaded cells which quantifies the rate of oligomer-induced dysregulation of intracellular Ca^2+^ homeostasis. We anticipate that the integrated single-molecule biophysics approaches highlighted here will develop further and in principle may be extended to the investigation of other protein aggregation systems under controlled experimental conditions.

## Highlights

• Method enables investigation of amyloid-β oligomer stoichiometry without requiring extrinsic fluorescent probes.
• Uses single-molecule stepwise photobleaching *in vitro*.
• Unveils heterogeneity within populations of oligomers.
• Assays oligomer-induced dysregulation of intracellular Ca^2+^ homeostasis in living cells.

## 1 Introduction

The amyloid-β peptide (Aβ) is derived via the proteolytic cleavage of the transmembrane amyloid precursor protein (APP) and ranges in length from 39 to 43 amino acids [1]. The longer peptides composed of 40- and 42- amino acids, so called Aβ(1–40) and Aβ(1–42), respectively, are observed in both healthy and Alzheimer’s disease (AD) brain tissue, with extracellular concentrations of Aβ estimated to be in the picomolar to low nanomolar range [2]. Intriguingly, this is several orders of magnitude lower than concentrations used in most studies designed to evaluate their structure, interactions and neurotoxicity. Mass-spectrometry based methods have, for example, recently estimated the concentration of Aβ(1–42) in human cerebrospinal fluid to be 1.5 nM [3], and the rate of Aβ production is approximated to be 2–4 peptides per second per neuron [4]. While concentrations of Aβ(1–42) an order of magnitude lower (0.1 nM–0.3 nM) have also been suggested to facilitate long-term potentiation, and recent evidence points towards a role for the Aβ(1–42) monomer as an antimicrobial peptide [5], [6], it is well established that Aβ(1–42) has a tendency to convert from a native and soluble form to an insoluble aggregated form, and that this conversion heavily influences cell viability [7], [8]. In the context of AD, Aβ(1–42) aggregation and neurotoxicity is widely considered to be a major hallmark.

**Fig. 1:**
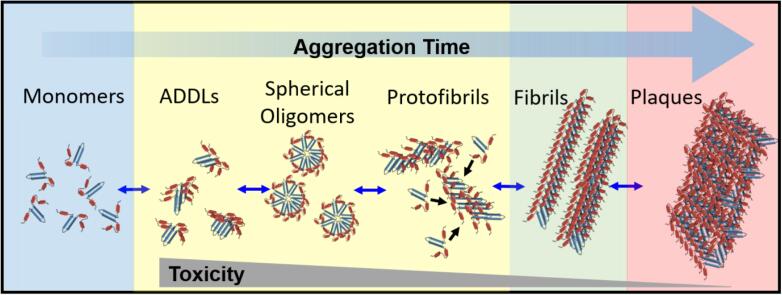
Schematic illustration of the time-dependent Aβ self-assembly process. Aβ monomers (blue) self-assemble into a series of low-mass oligomers (yellow), including amyloid-derived diffusible ligands (ADDLs), spherical oligomers and protofibrils, and high-mass fibrils (green) and plaques (red). While the precise time scales and morphological structures of Aβ aggregates are time-, concentration- and environment-sensitive, mounting evidence points to the low-mass oligomers as being the predominant neurotoxins. Not drawn to scale. (For interpretation of the references to colour in this figure legend, the reader is referred to the web version of this article.)

Aβ(1–40) represents the most abundant form of the peptide, comprising ~ 90% of the Aβ pool, while Aβ(1–42), comprises only 10% and contains two additional hydrophobic residues (isoleucine and alanine) at the C-terminus [9]. NMR-guided simulations of both structures suggest that this hydrophobic region is rigid due to the presence of β-hairpins formed by residues 31–34 and 38–41, and that this in turn may be partly responsible for a greater propensity of Aβ(1–42) to aggregate [8], [10]. Aβ self-assembly is highly dependent on environmental factors (including pH, temperature and ionic strength) and a variety of morphologies are known to co-exist along the aggregation pathway. These structures range from low-mass oligomers to the high-mass fibrils and plaques characteristic of an advanced AD brain [11], [12], [13], [14], [15] (Fig. 1), and although early research pointed towards fibrils and plaques as being the harmful species, focus has now shifted towards pre-fibrillar oligomers as being the predominant neurotoxins [16].

Oligomers are generally classified as Aβ-derived diffusible ligands (ADDLs) ranging from 12 kDa trimers to 108 kDa 24-mers [17], [18], spherical or annular oligomers of diameter 3–30 nm [19], and larger curvilinear protofibrils 4–11 nm in diameter and ≤ 200 nm in length [20], [21] (Fig. 1). While much has yet to be learned about the dynamic time-dependent assembly of the oligomeric species, they are believed to act as nucleation seeds for the oligomerization of Aβ monomers, and generally precede the formation of high-mass β -sheet rich fibrils and plaques. Although Aβ(1–42) has served as a test-bed for probing peptide aggregation *in vitro* [22], [23], [24], and mounting evidence now implicates Aβ oligomers in synapse impairment, reduced cognition and neuronal death [25], [26], the mechanisms through which single Aβ(1–42) peptides self-assemble and how the resulting structures confer neurotoxicity is still a hot topic of debate. One prevailing hypothesis suggests that Aβ(1–42) oligomers promote toxicity through a direct membrane disruption mechanism [27], with recent work implicating contact-induced dynamic perturbation of the cell’s mechanical properties as being an underlying physical factor [28]. Recent single-molecule approaches also indicate that small soluble Aβ(1–42) oligomers induce heightened levels of membrane permeabilization relative to larger species, though the magnitude of the disruption process varies between oligomeric forms [29]. Despite the lack of clarity on the mechanisms of toxicity, it is now abundantly clear that fibrillar and plaque-like aggregates correlate poorly with AD symptoms and that oligomers are orders of magnitude more harmful to living cells [30], [31], [32]. Understanding the mechanisms of Aβ oligomer assembly and neurotoxicity thus demands the development of experimental approaches capable of characterizing their stoichiometry, time-dependent heterogeneity and interactions under a wide range of environmental conditions, and, at physiological concentrations of the molecular reagents.

In recent years a number of structural techniques including cryo-electron microscopy (cryo-EM), electron diffraction, X-ray diffraction and solid-state NMR have provided high-resolution insights into Aβ assemblies either prepared *in vitro* or extracted from human brain tissue. Most of this work has involved the investigation of Aβ fibrils, and have broadly indicated that a complex interplay of bonding interactions, together with solvent and co-factor binding, structured cores and local disordered areas all contribute to their construction [33], [34]. Notably, recent cryo-EM studies on Aβ fibrils purified from human tissue have demonstrated that they are not only polymorphic but consist of similarly ordered protofilaments [35]. While such techniques have undoubtedly provided valuable structural insight, they generally either lack the ability to probe dynamic time-dependent changes and/or require non-physiological concentrations and conditions. In this context, the application of optical techniques based on absorption and fluorescence spectroscopy have been the most commonly used for aggregate detection and characterization [36], [37], [38]. For instance, the fluorescence intensity enhancement associated with small extrinsic molecules such as Thioflavin-T (ThT) [36] upon aggregate binding has become a workhorse tool for detecting a wide range of β-sheet rich Aβ aggregates *in vitro*. However, it is also well established that extrinsic probes may compete with aggregation inhibitors, which limits their use as screening tools [39] and suffer from a reduced affinity to the aggregated species at low pH due to local electrostatic repulsive effects [40]. Moreover, their mechanism of interaction is highly dependent on the formation of binding pockets along the aggregated structure, and their mode of binding has yet to be fully established [40], [41]. Consequently, extrinsic probes generally suffer from an inherent insensitivity to small oligomers that lack the necessary binding sites [42], [43]. In recent years, it has thus become abundantly clear that one of the fundamental barriers to progress in the fight against Aβ induced neurodegeneration arises from the intrinsic polymorphism of the oligomers, their time-dependent heterogeneity and the need to identify and selectively target distinct oligomeric species [44]. However, to complicate matters, additional challenges associated with investigating the details of a process as complex as Aβ oligomerization through optical microscopy methods arise from the fact that despite visible light enabling time-dependent dynamics to become accessible, a relatively poor spatial resolution is achieved compared to X-ray or electron microscopy. The problem here is that diffraction effects limit the spatial resolution to approximately the optical wavelength divided by twice the magnitude of the numerical aperture of the imaging system. For most setups, the numerical aperture is in the range 1.2–1.6, yielding a typical spatial resolution limit of ca. 180 nm for visible light of 500 nm. Furthermore, most biochemical assays involving extrinsic dyes rely on an average fluorescence signal from an assortment of large aggregates, oligomers and monomers, which masks heterogeneity within a sample population and fails to report on the presence of transient, intermediate and/or rare species. In this regard, single-molecule methods have emerged as a powerful means to characterize oligomeric species, one-at-a-time, to unveil the distribution of species present in solution, effectively revealing the type and relative abundance of oligomers that are otherwise hidden by the ensemble average. With the development of single-molecule approaches the behaviour of single Aβ oligomers can be probed with new levels of detail, greatly reducing the chance that such important information is bypassed [45]. Beyond these motivations, studying Aβ(1–42) self-assembly at physiological concentrations is a major hurdle for most biochemical assays, yet single-molecule detection techniques [46] are uniquely placed to overcome this challenge due to the intrinsic requirement for picomolar concentrations of fluorophores [47].

In recent years a number of single-molecule approaches have been employed to characterize oligomer size, growth, composition and interactions. For example, fluorescence imaging has been combined with near-infrared optical trapping to reveal the growth rate of single Aβ(1–42) species over the course of several hours [48]. Single-molecule Förster resonance energy transfer (smFRET) experiments, which quantitatively report on the separation distance between a donor and acceptor fluorophore over the range of approximately 1–10 nm, have also been used to reveal a broad distribution of interconverting conformations in Aβ(1–40) and Aβ(1–42) monomers with fluctuation timescales of order 35 ns [49]. Additional confocal-based FRET-sensing approaches involving N-terminally labelled Aβ(1–42) and synthetic liposomes incorporating TRITC in the lipid bilayer region have provided dynamic and temporal insight into oligomer-membrane interactions and when combined with fluorescence correlation spectroscopy, the method has enabled detection of extremely low stoichiometry populations of oligomers containing < 10 Aβ(1–42) peptides [50]. Another confocal-based strategy that has enabled the rapid detection of oligomers composed of two different labelled Aβ peptides in solution utilizes two-colour excitation and the detection of coincident fluorescence bursts. For example, converged blue and red lasers have facilitated identification of Alexa Fluor 488- and Alexa Fluor 647-labelled Aβ(1–40) and Aβ(1–42) monomers as they pass through the confocal volume, while also enabling detection of mixed-label species [51], [52]. However, in many such confocal-geometry based experiments, the observation time window is limited to only the few milliseconds the labelled aggregate takes to diffuse through a confocal volume, and given a heterogeneous distribution of oligomers with different diffusive properties, the fraction of molecules that is actually analysed depends strongly on the measurement time, the excitation intensity and the size of the reservoir associated with each freely diffusing species.

Recent additions to the array of single particle techniques applicable to the study of amyloidogenic assembly includes interferometric scattering mass spectrometry (iSCAMS), whereby the scattering intensity obtained from single biomolecules in solution is used to quantify mass. In this scenario, the scattering signal is proportional to the peptide mass and has been used to identify the binding of low-order oligomers composed of relatively large (66.5 kDa) molecular weight proteins [53]. However, in the context of much lower molecular weight species implicated in neurodegeneration, the technique is currently unable to determine the stoichiometry of small nucleating oligomers, evaluate individual assembly steps and has the intrinsic requirement for low-scattering *in vitro* conditions.

**Fig. 2:**
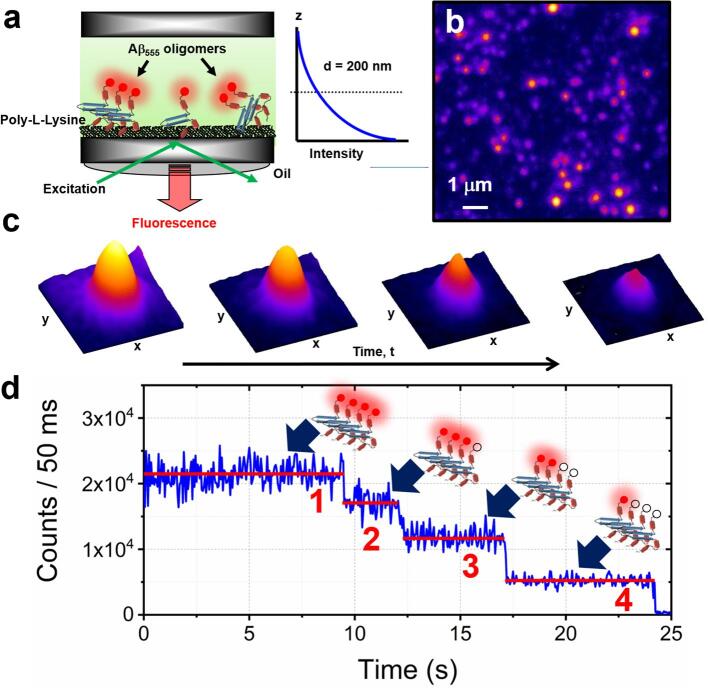
Overview of the single-molecule stepwise photobleaching method to evaluate oligomer stoichiometry. (a) Wide-field single-molecule imaging techniques such as total internal reflection fluorescence (TIRF) microscopy utilize a high numerical aperture objective lens to totally internally reflect excitation light at the boundary between a glass coverslip and sample, forming an evanescent field which decays exponentially into the sample. Typical penetration depths, Δ, are ca. 100–200 nm. In this case, only immobilized oligomers, composed of labelled peptides are stimulated to emit fluorescence, minimizing background fluorescence from the ensemble solution. (b) A representative wide-field image of immobilized and spatially separated oligomers composed of Aβ(1–42) peptides labelled at the N-terminus with the fluorescent molecule HiLyte Fluor 555 (λ_ex_ = 532 nm). (c) A representative false-colour point-spread function obtained from a single immobilized oligomer as a function of time displays a stepwise decrease in integrated signal intensity, enabling the number of monomeric subunits to be counted. In the fluorescence trajectory shown in (d), the presence of four discrete stepwise photobleaching events corresponds to the identification of a tetramer.

Of all single-molecule based techniques, the stepwise-photobleaching method, based on the long-term imaging of tracked biomolecules, has been the most widely implemented for assessing biomolecular size distributions [47], [54], [55], [56], [57] (Fig. 2). Photobleaching takes place when the excited fluorophore permanently loses its ability to fluoresce due to photon-induced chemical damage and covalent modification. While the average number of excitation and emission cycles that take place prior to photobleaching is dependent on the fluorophore structure and local environment, and the rate of photobleaching can be altered by modification of the excitation intensity, the complete loss of fluorescence is exploited to unambiguously determine the number of peptides-per-oligomer. Here, each Aβ peptide carries a single fluorescent molecule, and upon excitation, each label within an immobilized oligomer photobleaches, providing a stepwise fluorescence trajectory in which each step in a sampling time window corresponds to the bleaching of an integer number of fluorophores, typically with several single molecule steps in the case of low stoichiometry complexes and especially towards the end of a photobleach time course [58]. By counting the number of photobleaching steps, in a probabilistic manner, it is therefore possible to identify the number of peptides-per-oligomer (Fig. 2). The method involves the direct counting of all single molecule photobleaching steps and is typically limited to the detection of 10-mers, beyond which the trajectories become exponential-like and require other analytical approaches such as Fourier spectral analysis [59], but nevertheless the direct single step counting method has been employed by a number of groups in the field. For example, the stepwise photobleaching of Aβ(1–40) peptides labelled with fluorescent dyes at the N-terminus has been used to characterize the distribution of oligomers on supported lipid bilayers [60], naked glass coverslips [54] and polyethylene glycol-coated substrates [47]. These systems are of course highly artificial, each have their own practical limitations with regards to substrate compatibility, and they may influence Aβ-surface binding, meaning caution should be taken with the interpretation of data in the context of a physiological environment. However, such *in vitro* systems offer an excellent opportunity to explore the effect of environmental conditions on Aβ oligomerization within a controllable, tuneable and water-soluble environment in the absence of the many extraneous biochemical processes found in vivo. In the latter case, picomolar concentrations of FAM-labelled Aβ(1–40) monomers were shown to oligomerize into a series of dimers, trimers, tetramers and pentamers in the presence of acidic conditions and zinc coordination. The technique has even extended to the detection of Alexa Fluor 647-labelled cys-Aβ(1–42) in human cerebrospinal fluid [61]. In all cases, the total fluorescence intensity obtained for each oligomer also scales with the number of peptides, enabling a second optical fingerprint to be obtained from the same experiment. Clearly this method holds substantial promise for the quantification of Aβ oligomer size distributions and has already been demonstrated to operate over a wide variety of experimental conditions.

In this work, we describe the implementation of a wide-field, objective-based, total internal reflection fluorescence microscopy imaging method to access the stepwise-photobleaching trajectories obtained from single surface-immobilized oligomers composed of Aβ(1–42) peptides labelled at the N-terminal position with HiLyte Fluor 555 (Aβ_555_), a member of the cyanine family of fluorescent compounds. Given that soluble oligomers formed at different stages of the aggregation process have been shown to induce permeabilization of the lipid bilayer, we also describe a ratiometric fluorescence assay that provides a quantifiable real-time metric of intracellular Ca^2+^ levels in response to the injection of pre-formed and robustly characterized Aβ_555_ oligomer populations.

In the sections that follow, we describe a series of procedures that enable Aβ_555_ oligomer stoichiometries to be measured via the quantized stepwise photobleaching method, which can then be correlated against orthogonal data from a ratiometric assay for quantifying Aβ_555_ activity using a live-cell approach, such that the integration of these two approaches provides far more insight than using the approaches in isolation. We also describe the process of data acquisition and analysis, provide representative data sets, and identify troubleshooting hints.

## 2 Methods

### 2.1 Aβ_555_ preparation and handling

**Fig. 3:**
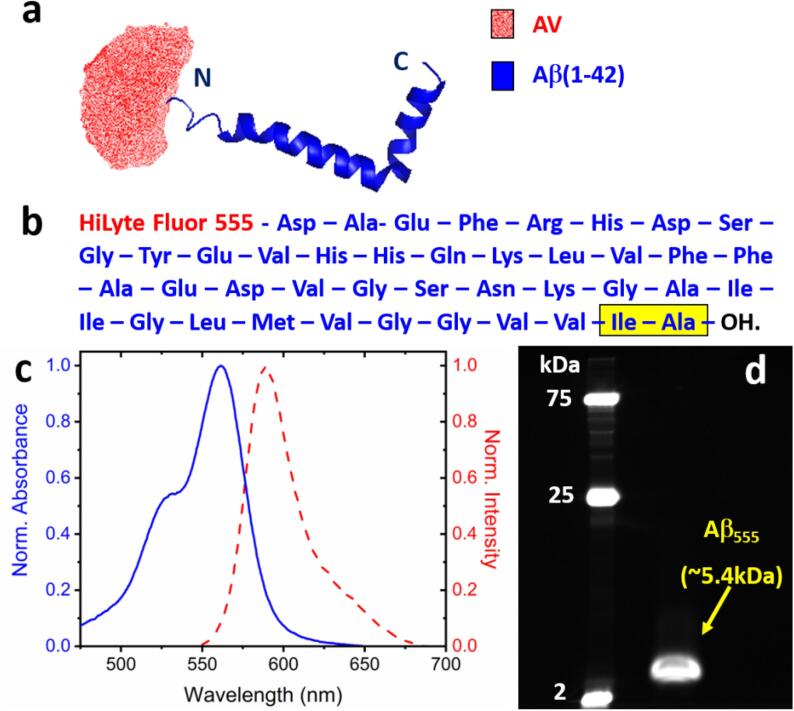
Schematic of the Aβ_555_ peptide used for single-molecule photobleaching studies. (a) Aβ(1–42) (blue, PDB ID = 1IYT)[67] is labelled at the N-terminus with HiLyte Fluor 555. The accessible volume of the dye is illustrated by the semi-transparent red surface and was calculated using a freely available structural modelling algorithm [68] in which a linker length of 13 Å was used and the dye was modelled as a single ellipsoid with radii R_1_ = 7.1 Å, R_2_ = 11.1 Å and R_3_ = 4.9 Å as estimated with ChemDraw after energy optimization with MMFF94 minimization. (b) The sequence of Aβ_555_. Hydrophobic residues at the C-terminus are highlighted in yellow. (c) Normalized absorption (blue solid line) and fluorescence emission (red dashed line) spectra of Aβ_555_ in 50 mM Tris buffer (pH 8). (d) Fluorescence image of denaturing SDS-PAGE gel of Aβ_555_ immediately after the preparation process indicates a lack of pre-formed high-mass aggregates.

Lyophilized Aβ_555_ peptides were purchased from Cambridge Bioscience (UK) (part number ANA60480-01). N-terminal attachment of the Aβ peptides to HiLyte Fluor 555 (Fig. 3a, b) was performed by the manufacturer and purification was characterized using mass spectroscopy and high-performance liquid chromatography. HiLyte Fluor 555 is a derivative of the widely used Cy3 dye which has absorption and fluorescence emission peaks at 551 nm and 567 nm, respectively (Fig. 3c). Despite exhibiting a shorter Stokes shift relative to Cy3, the molecule contains a long chain linking both aromatic groups which minimizes flexibility and reduces the probability of photoblinking. To prepare Aβ_555_ for analysis, it is first important to monomerize the lyophilized stock. This can be achieved by dissolving the lyophilized peptides directly from the manufacturer in hexafluoroisopropanol (HFIP) (Sigma Aldrich, part number 105228) to a concentration of 0.2 gL^-1^. Once dissolved, the solution can be aliquoted into 10 μL volumes in Protein LoBind tubes (Eppendorf, Part Number 0030108116) in accordance with well-established procedures used by us and many others [24], [62], [63], [64], [65]. Aliquots should then be desiccated under vacuum for 5–6 h at room temperature to remove HFIP, and stored at −20 °C prior to use.

SDS-PAGE can be employed to assess whether the procedure is successful at producing mostly monomeric species. In this regard, we used a self-poured 15% acrylamide Tris-glycine protein gel (10 well, 30 μL per well). Here, 2 μg of aliquoted Aβ_555_ was suspended in 4 μL of anhydrous dimethyl sulfoxide (DMSO) (Sigma Aldrich, part number 276855) and subsequently diluted in 1 × SDS loading buffer (50 mM Tris, pH 6.8, 2% (w/v) SDS, 0.05% (w/v) bromophenol blue, 10% (v/v) glycerol and 5% (v/v) β- mercaptoethanol). Gels were then run at 220 V for 75 min. A pre-stained molecular weight marker (Precision Plus Protein™ Dual Xtra Prestained Protein Standards, Bio-Rad) was used to determine the end-point of the electrophoresis and fluorescence images of the gel were captured using a Gel-Doc system (Bio-Rad). Since Aβ(1–42) is known to produce SDS-resistant aggregates [66], we used the gel to visualize the abundance of species present in solution. Immediately after preparation, only a single band consistent with the presence of monomeric Aβ_555_ at ~5.4 kDa was observed (Fig. 3d).

### 2.2 Aβ_555_ oligomer preparation

A number of well-established procedures may be followed to produce low-mass oligomers *in vitro* [24], [52], [69], [70], [71]. However, for the purposes of proof-of-principle of this method, we were interested in the preparation of small globular oligomers. These can be prepared by suspending freshly prepared Aβ_555_ monomers in 1–5 μL of DMSO and thereafter immediately diluting in aggregation buffer to a final Aβ concentration of 1 – 10 pM. We use 50 mM Tris buffer (pH 8) containing ≤4% (v/v) HFIP and <1% (v/v) DMSO as the aggregation conditions to stimulate oligomerization. While high concentrations of HFIP have been popular agents for the monomerization of peptides, low concentrations in the range 1–4 % (v/v) are known to rapidly promote the formation of Aβ oligomers [24], [70] via a mechanism involving the formation of HFIP micro-droplets in solution that act as interfaces to promote aggregation. Although it is not yet clear if these oligomers represent a biologically relevant morphology in the context of Alzheimer’s disease pathology, the acceleration of Aβ aggregation has already been observed at the interface of biomimetic membranes [72]. Additionally, highly fluorinated compounds, similar to HFIP, have been shown to promote the formation of Aβ structures that may be related to the appearance of cognitive defects [73]. The timescale of 1–2% (v/v) HFIP-induced aggregation of Aβ(1–40) has also been shown to rapidly reduce from weeks to minutes [71], motivating us to examine Aβ(1–42) oligomerization under similar working conditions.

### 2.3 Microscope flow-cell fabrication and immobilization of Aβ_555_ oligomers

**Fig. 4:**
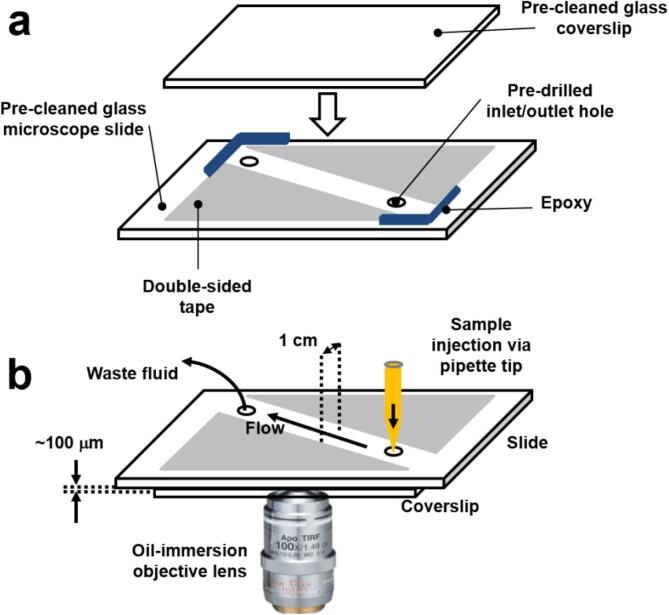
A schematic illustration of the flow-cell manufacturing process. (a) Double sided tape is placed on a pre-cleaned glass microscope slide containing 1 inlet and 1 outlet hole for sample injection. A pre-cleaned glass microscope coverslip is added directly on top of the double-sided tape to form a chamber. (b) After coating the interior of the chamber with poly-l-lysine and the injection of pre-formed Aβ_555_ complexes, the fluidic chamber is placed coverslip down onto an oil immersion objective lens housed in an inverted microscope to enable monitoring of surface immobilized oligomers.

Single-molecule stepwise photobleaching trajectories were obtained from Aβ_555_ oligomers immobilized onto the surface of a pre-cleaned glass coverslip (22 × 50 mm, No. 1.5 thickness, Scientific Laboratory Supplies Ltd, part number MIC3246), which formed part of a ~1 cm wide microscope flow-cell. A schematic illustration of the flow-cell is shown in Fig. 4. The text that follows provides a step-by-step guide to fabricating flow-cells and performing oligomer immobilization.

To begin the process of preparing a flow-cell, first drill two holes (ϕ = 1 mm) in 25 × 75 × 1 mm glass microscope slides (Polysciences Inc., part number 22245) for the purposes of sample injection into the flow-cell via pipette tip as schematically shown in Fig. 4a. It is worth noting that flow-cells were typically prepared in batches of five. Small diamond core drill bits (Eternal Tools, UK, part number SDCD1100) were used to drill 1 inlet and 1 outlet hole per slide. Prior to flow-cell fabrication, each pre-drilled microscope slide and glass coverslip must then be thoroughly cleaned as per steps 1–3 below:

1) To begin the cleaning process sonicate each of the pre-drilled microscope slides and coverslips in the following solutions sequentially: 2% Hellmanex III, 98% ultra-pure water solution (15 min), ultra-pure water (5 min), acetone (15 min), ultra-pure water (5 min), 1 M potassium hydroxide (15 min), ultra-pure water (5 min), methanol (15 min), ultra-pure water (5 min), 1 M potassium hydroxide (15 min), ultra-pure water (5 min). After each solvent step, rinse the slides and coverslips thoroughly with ultra-pure water.
2) Slides and coverslips can be washed one day and refreshed the next. To refresh, sonicate each slide and coverslip sequentially in ultra-pure water (5 min), 1 M potassium hydroxide (15 min) and ultra-pure water (5 min). After the wash step involving potassium hydroxide, the slides and coverslips must be rinsed thoroughly with ultra-pure water. Slides and coverslips are stored for up to one week in ultra-pure water after cleaning if refreshed daily.
3) When ready to be used, gently dry each slide and coverslip using filtered nitrogen gas and remove any residual water droplets.

Next, fabricate flow-cells by applying double-sided tape to the pre-cleaned microscope slide as demonstrated in Fig. 4a in order to construct the walls of the chamber. Care should be taken to ensure a channel thickness of ~1 cm. Apply a pre-cleaned glass coverslip on top to form the ceiling of the chamber and gently push it against the tape using a pipette tip to complete fabrication. Remove residual tape carefully with a razor blade and seal the edges of the chamber using epoxy adhesive (Araldite, part number ARA-400012).

Note that it is possible to re-use glass microscope slides from flow-cells after use. Removal of used coverslips, double-sided tape and epoxy may be achieved by submerging the used chambers in acetone for 1–2 h, then carefully removing the coverslip, epoxy and tape from the slide using a razor blade. After removal, slides should be sequentially scrubbed with optical tissue soaked in acetone and methanol until visibly clean, before being subjected to the cleaning procedure outlined in steps 1–3. Depending on the usage, the slide cleaning procedure for re-used slides may require an additional etching step prior to steps 1–3. In this case, pre-scrubbed slides and coverslips can be transferred to a fume hood and submerged in a 50:50 mix of hydrogen peroxide and hydrochloric acid for 1–2 h. Slides should be left for no longer than 2 h. Etched slides must then be thoroughly rinsed with ultra-pure water prior to the cleaning procedure.

In order to immobilize pre-formed Aβ_555_ oligomers or freshly prepared Aβ_555_ peptides, first coat the flow-cell with 60–100 μL of 0.1% poly-l-lysine (MW = 150–300 kDa, Sigma Aldrich, part number P8920). Flush the solution into the flow-cell using a pipette tip injected into an inlet hole and incubate the chamber for 15 min at room temperature. Attachment of biological molecules to glass surfaces has frequently been accomplished via electrostatic interactions with poly-L-Lysine [74], [75] and in this case, we utilize the approach to bypass the need for any additional functionalization of the peptide. Here, the negative surface charge associated with Aβ(1–42) enables the positively charged poly-l-lysine polymers to become effective for non-specific immobilization. After 15 min, rinse each flow-cell with 3 × 100 μL of buffer to remove any unbound polymers. Following the wash step, flush 60–100 μL of freshly prepared Aβ_555_ or pre-formed Aβ_555_ oligomers at 1–10 pM onto the poly-l-lysine coated surface. It is important to note that the Aβ_555_ solutions must not be diluted prior to being flushed into the chamber, as serial dilutions may alter the final aggregated state. The flow-cell is now ready to be imaged by total internal reflection fluorescence (TIRF) microscopy.

### 2.4 Microscope Setup: Objective-based total internal reflection fluorescence microscopy

**Fig. 5:**
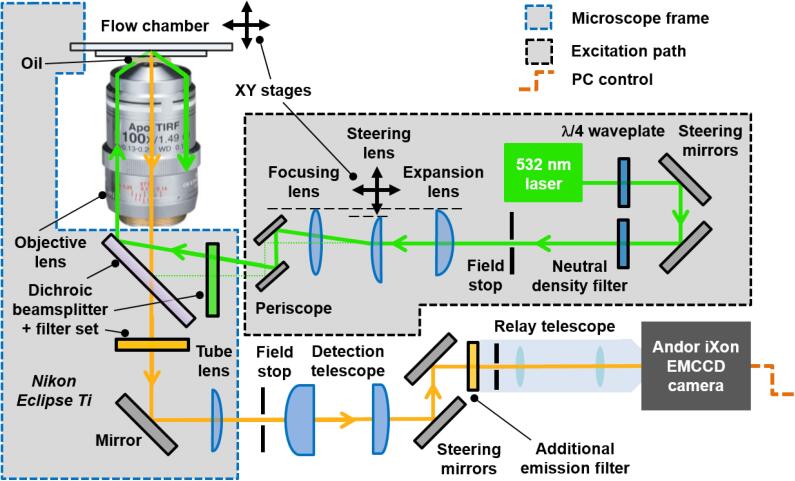
A schematic illustration of the objective-based TIRF microscope used for stepwise photobleaching experiments. The excitation source and optics are shown. Detailed descriptions of all optical components are found in the main text. The dotted green line represents the excitation path in epi-illumination mode. The figure is for schematic illustration only and is not drawn to scale.

For single molecule photobleaching experiments, we utilized a custom-built objective-based TIRF geometry for fluorophore excitation. Fig. 5 shows a schematic illustration of the microscopy optics. All lenses, translation stages and mirrors (part number BB1-E02) were purchased from Thorlabs Inc., USA unless otherwise specified. All optical components had anti-reflection coatings for optical wavelengths in the range 350–700 nm.

In our setup, a TEM_00_ continuous wave 532 nm excitation line (Coherent Ltd., UK, OBIS 532 nm LS 50 mW part number 1261780) was circularised with a quarter waveplate (Thorlabs, AQWP05M-580) and expanded with a 3 × telescope (comprised of expansion lens: f = 100 mm planoconvex LA1509-A-ML and steering lens: f = 300 mm achromatic doublet AC254-300-A-ML), with the latter mounted on an x-y translation stage (part number LX20/M). We note that one is able to select from a wide variety of solid-state, diode, or gas lasers to optimally match the illumination wavelength to the absorption spectra of the fluorescent probe, and in principle the maximum excitation wavelength is 550 nm. In this work we used an optically pumped semiconductor laser at 532 nm to excite HiLyte Fluor 555 at 55% of maximum absorption, though 543 nm or 546 nm lines could offer further optimization. The expanded beam was focused using a third biconvex lens (part number LB1779-A-ML) and directed by periscope (part number RS99) onto the back aperture of an oil-immersion objective lens (Nikon CFI Apo TIRF 100x NA 1.49, part number MRD01991) housed within an inverted fluorescence microscope (Nikon Eclipse T*i*) such that the size, location and illumination scheme could be easily altered if needed, for example with a neutral density filter wheel (part number FW1AND). The laser was focused onto the back aperture of the objective lens such that a collimated beam incided onto the internal coverslip face of the flowcell at an angle beyond the glass-water critical angle, thus sustaining total internal reflection. The coverslip was optically coupled to the objective lens by a thin layer of fluorescence-free objective immersion oil (Olympus, part number IMMOIL-F30CC). The emission was then separated from the laser line with a filter cube (Chroma Technology Corp., part number 59907-ET-532/640 nm Laser Dual Band Set, including excitation filter, dichroic beamsplitter and emission filter).

After the filter cube, mirror, and f = 200 mm tube lens within the microscope body, the detection path exited the microscope frame at the lower side port. Another 3 × telescope (comprised of achromatic doublet lenses AC254-50-A and AC254-150-A with f = 50 mm and f = 150 mm, respectively) was then used to magnify the image. In turn this magnified image was relayed onto an EMCCD camera (iXon Ultra 897, Andor Technology Ltd.). The particular implementation used a 1:1 commercial DV^2^ relay telescope from Photometrics Inc, albeit set to bypass the colour splitting function. This image magnification corresponds to an object-plane pixel size of 50 nm on the camera, or roughly 15 pixels per diffraction limited spot.

In order to build the objective-based TIRF microscope, the following procedure should be adopted. First, the excitation laser needs to be aligned to the centre of the microscope field of view. Steps 1–5 below provide a practical guide to achieving this. Refer to safety considerations at the end of this procedure before attempting any alignment process.

**Fig. 6:**
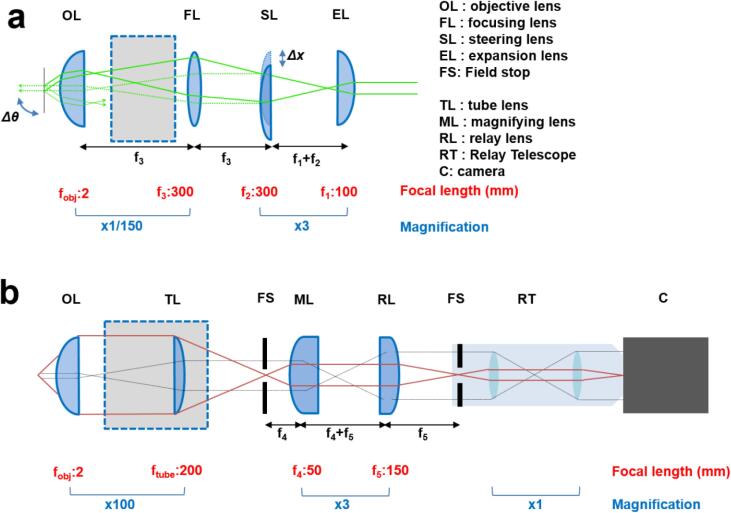
Ray optics diagrams of the objective-based TIRF microscopy setup. Ray diagram and adjacent optical elements in the (a) excitation and (b) detection paths are shown. Focal lengths (red text) and magnifications (blue text) associated with each component are also shown.
  The ratio of the two focal lengths (f_2_/f_1_ = 3) determines the magnification of the beam, in this case to expand the beam from an initial diameter (at 1/e^2^ intensity) of 1.3 mm at the expansion lens, to a diameter of 3.9 mm at the focusing lens. Together with the ratio of the second telescope formed by the focusing lens and objective (f_obj_/f_3_ = 1/150) (Fig. 6a) this gives the expected size of the illuminated region in the field of view (d = 3.9 mm / 150 = 26 µm).
(5) Note the Vernier scale position of the lateral micrometer on the steering lens and move this to the nearest reproducible mark. Record this position as x_o_. Readjust the periscope mirrors to compensate the laser movement. The microscope is now in a crudely calibrated narrowfield fluorescence configuration and the vertically-propagating laser can be used as a reference for the central axis of the objective.
  The next goal is to align the detection optics (steps 6–9).
(6) Project a diffuse light source (such as a brightfield lamp and condenser) through the objective and tube lens to produce a broad beam of light from the side port. Place and align the steering mirrors to transmit this beam onto the camera via the relay telescope, leaving enough room near the port for the magnifying telescope lenses (>f_4_ + f_5_ = 200 mm). The second relay lens must be set a focal length (f_5_) away from the entrance of the relay telescope (Fig. 6b).
(7) Place a droplet of immersion oil on the top of the objective lens. Obtain a pinhole whose size is roughly double the expected laser size (e.g. d = 50 µm, part number P50D). Secure the pinhole to a microscope slide with which its position can be controlled using the microscope stage. Adjust the pinhole until the laser emerges with maximum transmitted power. Disable the laser but do not move the pinhole.
(8) Activate the camera software of choice. Under brightfield illumination, finely adjust the detection steering mirrors, walking the image until the pinhole is centred and focused within the camera’s field of view.
(9) Place the magnification lenses in one after the other, separated by f_4_ + f_5_ = 200 mm (Fig. 6b), adjusting each until the scene reappears and the pinhole is recentred, this time with the correct magnification. Alternatively, use field stops at the positions where the magnification lenses will be placed to ensure these are centred on the field of view. Both excitation and detection paths are now crudely aligned with one another.
  Next, locate fluorescence emission from bright microspheres arising from the illumination spot at the centre of the field of view and perform final fine-tuned alignments of the excitation and detection optics as documented in steps 10–17. For photobleaching experiments only single-colour illumination and detection is required.
(10) Prepare a dilute (~0.002%) solution of red fluorescent microspheres of diameter 0.2 μm (Thermo Fisher Scientific, Part Number F8810) in 50 mM Tris, 100 mM NaCl buffer (pH 8.0). Add the solution to a pre-constructed flow chamber and incubate for 10–15 min at room temperature. After incubation, flush the chamber with buffer to remove any microspheres unattached to the glass coverslip.
(11) Place a droplet of immersion oil on the top of the objective lens and mount the flow chamber containing the microspheres on top.
(12) If the laser beam / fluorescent beads do not appear in the field of view, set the lateral micrometer to the calibrated narrowfield position x_o_. Then, find the position of the laser on the field of view by moving the lower periscope mirror.
(13) Complete the narrowfield (zero angle of incidence) alignment by redirecting the laser i) through the centre of the objective lens (i.e. in the projection) using the lower periscope mirror and ii) back onto the camera field of view with the upper periscope mirror. Iterate i) and ii) until the laser is centred in both planes.
(14) Image the fluorescent microspheres on the camera and focus being careful to use a low electron multiplication gain. An integration time of 50–100 ms will suffice for imaging purposes.
(15) For a robust alignment in which the beam’s angle of incidence can be varied continuously without the beam ‘walking off’ the field of view, the excitation steering lens must sit at the correct distance from the focusing lens. Achieve this condition by dithering the lateral micrometer whilst smoothly varying the longitudinal position of the steering lens, until the dithering can no longer be seen on the camera as a change in beam position but only in angle. Check this is correct by moving the micrometer to its extremes; the projection of the beam on the sample should become increasingly elliptical as the angle of incidence increases, but its centre should not move. Walk the periscope mirrors to recentre the beam in the field of view.
(16) Translate the micrometer until the beam surpasses the glass-water critical angle and the excitation becomes restricted to the interface. Refocus to image this layer; only microspheres sufficiently close enough to the glass coverslip will yield signal. The surface signal reaches a maximum relative to the background, before quickly clipping/disappearing as the beam is translated off the rear of the objective lens. This condition of maximum signal to background ratio results from the standing-wave amplification and signifies the optimal TIRF that can be achieved using the setup.
(17) Most commercial excitation lasers are linearly polarised, as in this case. The polarisation can be checked by rotating a linear polarising film (such as LPVISE2X2) in the beam at low power (~1mW, making use of the neutral density filters as necessary) and detecting extinction along a particular axis. Insert the λ/4 waveplate in a rotating mount (RSP1D/M) into the excitation path near the laser head. To circularise the output beam, the waveplate should be rotated such that the input polarisation axis is halfway between the fast and slow axes of the waveplate. If fluorophores near the surface have a fixed or preferred orientation, particularly along the direction of beam inclination, the input polarisation may have a modest effect on the output signal. If necessary, adjust the quarter waveplate to minimise signal anisotropy.

(1) Before firing the laser, fix the laser head facing away from and offset from the microscope input port (Fig. 5). Set up the periscope in front of the port, with the upper mirror directed into the port and the lower mirror facing away. Set up the steering mirrors and neutral density filter wheel in line with the laser head, such that the mirrors each turn the beam through an approximate right angle and the output beamline points towards the lower periscope mirror. Unscrew the objective. At an eye safe laser power (<1mW at source), adjust the mirrors and periscope to ‘walk the beam’ by counter-adjusting the mirrors until the beam emerges centrally from the objective turret. Disable the laser and fix the mirrors in place.
(2) Assemble the focusing lens onto an x-y adjustable mount and place at a focal length (f_3_ = 300 mm) from the back aperture of the objective lens along the optical axis. Replace the objective and, with the beam visible, adjust the focusing lens until the beam again emerges centrally and collimated from the objective, such that the beam projection is symmetrical and minimal in size. Turn the laser off.
(3) Mount the steering lens in an x-y adjustable mount on the linear translation stage. Set this at a distance upstream from the focusing lens, equal to the focal length of that focusing lens (f_3_ = 300 mm). Ensure that the curved face of the steering lens is facing toward the focusing lens. Turn the laser on and adjust this mount until the beam is centred both on the lens and through the objective; the beam centre may appear very diffuse, in which case adjust for maximum power through the objective. Failing this, turn the laser off and unscrew the focusing lens making sure to retain its position, and retry adjusting the steering lens with the laser on. Then turn the laser off and replace the focusing lens.
(4) Starting from the steering lens position, place the expansion lens further towards the laser source at a distance equal to the sum of the focal lengths of the expansion lens (f_1_ = 100 mm) and of the steering lens (f_2_ = 300 mm; i.e. f_1_ + f_2_ = 400 mm) (Fig. 6a). Point the curved face of the expansion lens toward the source to minimize aberrations in the beam. As before, turn the laser on and adjust this lens to centre and collimate the beam output from the objective.

#### 2.4.1 Angular calibration

**Fig. 7:**
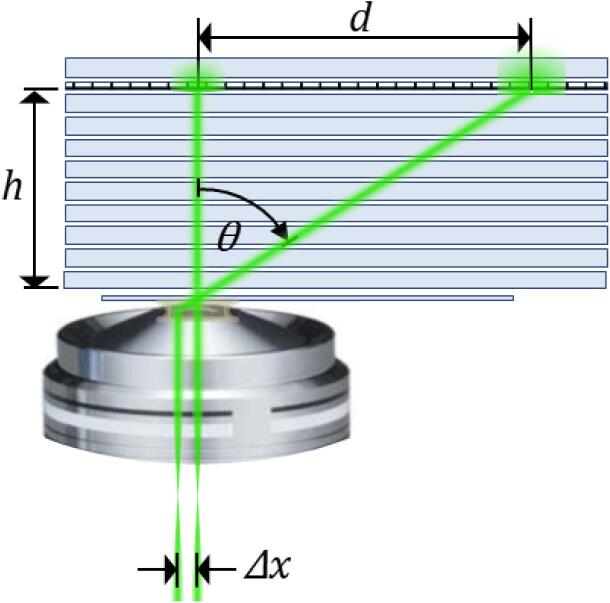
Schematic of the slide stack procedure to calibrate the angle of the beam by measuring its projected displacement as a function of steering lens position.

Once robust alignment is established, the lateral position of the steering lens can be used to build a look up table for the beam angle at the front aperture and the corresponding penetration depth. Then, any beam angle in the experimentally accessible range can be dialled, even during an experiment and without changing the sample. Our approach requires a prism with refractive index matched to the objective immersion medium. For oil immersion objectives, this is simple to construct by sandwiching 5–10 pre-cleaned microscope slides together to form a slide stack, with copious amounts of immersion oil on each slide to exclude air gaps. Compress the slide stack and remove excess oil from the sides. Measure the height of the stack to <0.1 mm precision with callipers; this should lie in the range 6–12 mm. Cut a 25 × 75 mm piece of high quality graph paper with precise and fine (e.g. 1 mm) graduations, soaking it in immersion oil. Secure it on top of the stack with an extra slide. Finally, finish the slide stack by sealing the sides with electrical insulation tape. A schematic illustration of the slide stack is shown in Fig. 7.

Move the steering lens micrometer to the narrowfield position (x_o_ = 3.50 mm in this case) and ensure the laser is vertically aligned along the axis of the objective. Attach a coverslip to the underside of the stack (away from the graph paper) using a drop of oil. Then apply oil to the microscope objective and place the stack on the microscope stage, graph paper upwards. Activate the laser, which should project a spot on the paper along the axis of the objective. Move the stage towards one end of the slide, such that a division of the graph paper intersects the centre of the laser spot. Focus on the internal surface of the coverslip as usual. Then, without moving the stage, shift the steering lens micrometer in fixed steps and record the displacement of the laser spot, d*,* as a function of micrometer position x. The uncertainty in displacement can be estimated as the semimajor axis of the spot.

**Fig. 8:**
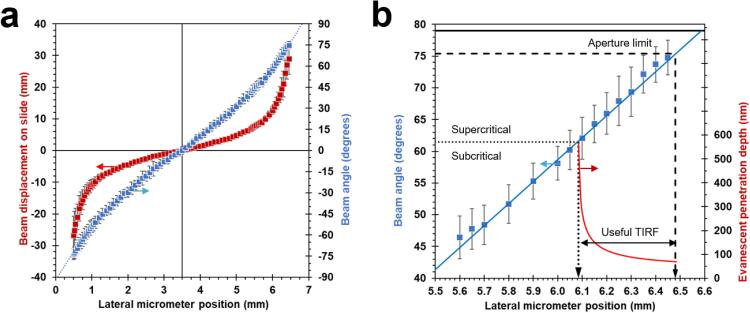
Exemplar data for beam angle calibration measurements. (a) Beam displacement on a slide stack as a function of the lateral position of the steering lens (red) and the corresponding angle of the beam emerging from the objective front aperture (blue) fitted with cubic function to model slight positive spherical aberration (blue dotted line). Note the narrowfield conditions (*θ, d* = 0) is aligned after the micrometer offset is specified at x_o_=3.50 mm. (b) Detail of the angle-micrometer curve (blue) with local linear least squares fit annotated with the theoretical glass-water critical angle, *θ_C_* ≈ 61°, (dotted black line), the maximum shift admitted by the objective back aperture, *θ*(*x_max_*) ≈ 75° (dashed black line), and the marginal ray at the front aperture, *θ_NA_* = *asin* (*NA*/*n_oil_*) ≈ 79° (solid black line). Also shown is the theoretical penetration depth, d, (red) across the accessible range of supercritical angles. The centre of the projected spot was determined on the linear slide scale and its error given by half the length of the projected spot.

At high displacements the beam projection becomes increasingly elongated and eventually the beam walks off the back aperture. Since we use steering and focusing lenses of the same focal length, this limit is expected to occur when the steering lens micrometer is shifted by half the objective back aperture, such that $x_{\max}\approx x_{o}+NA\cdot f_{obj}$, (in our case 6.48 mm = 3.50 mm + 1.49 × 2 mm). This expectation is close to what we observed as shown in Fig. 8.

The angle *θ* at which the beam emerges is related to the displacement *d* by the simple geometric relation $\theta =atan\left(d/h\right)$ where *h* is the stack thickness. For sub-critical angles $\theta \leq \theta_{C}$, the beam is refracted again at the glass-sample interface according to Snell’s law. However, for super-critical angles $\theta >\theta_{C}$, only the evanescent wave associated with TIRF exists. The magnitude of the penetration depth, Δ, associated with the evanescent wave may be estimated using the equation

$$\Delta =\frac{\lambda }{4\pi \sqrt{n_{g}^{2}sin^{2}\theta -n_{w}^{2}}}$$

where $n_{g}$ is the refractive index of the glass coverslip, $n_{w}$ is the refractive index of the sample and λ is the excitation wavelength. In this case, Δ is a decreasing function of the angle of incidence, θ, and is typically 100–200 nm.

#### 2.4.2 TIRF safety considerations

With respect to safety, it is important to fully enclose the excitation path to avoid exposure. In addition, we make use of black laser curtains surrounding the optical bench, and both a laser interlock and ‘laser in use’ warning sign to inform others that they must take necessary precautions before entering the area. Appropriate laser safety goggles should be worn and any laser shutters should be kept closed when the excitation source is not required. Finally, one should never look directly into the light path when the shutter is open.

### 2.5 Stepwise photobleaching data Acquisition: A general imaging protocol to determine oligomer stoichiometry.

**Fig. 9:**
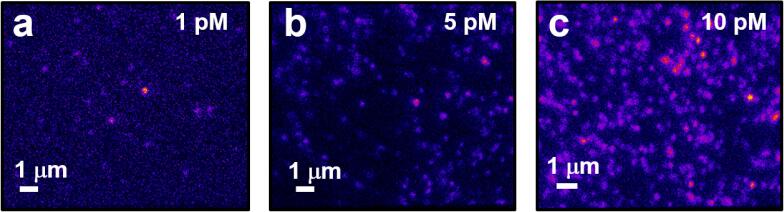
Representative TIRF images of immobilized Aβ_555_ as a function of peptide concentration. Wide-field fluorescence images of Aβ_555_ immobilized onto a poly-l-lysine coated glass coverslip at concentrations of (a) 1 pM, (b) 5 pM and (c) 10 pM.

The data we collect from the objective-based TIRF system is integrated fluorescence intensities from non-specifically immobilized Aβ_555_ oligomers. Immobile oligomers were imaged in 25 × 25 μm regions, and plots of fluorescence intensity generated as a function of position. For each field of view, fluorescence emission from ~ 150–200 immobilized oligomers were acquired in parallel over a time-course of typically 30 s using an EMCCD integration time of 50 ms. To provide confidence that fluorescence signals were representative of single Aβ_555_ peptides or oligomers, varying concentrations (1 – 10 pM) were added to the surface, resulting in a corresponding increase in the number of fluorescent spots per field of view as shown in Fig. 9 and a four-fold decrease in the mean nearest-spot separation distance.

An excitation power of 2 mW, as measured immediately prior to entering the objective lens, was selected to promote dye photobleaching over the timescale of the measurement. Under these conditions, monomer signal-to-noise ratios were typically in the range 3–5. Excitation was continued until fluorescence emission dropped to background levels and at the end of each 30 s collection period, >90% of all fluorescent spots had completely photobleached. In order to construct fluorescence-versus-time trajectories from immobilized oligomers, the integrated point-spread function signals were extracted from the sequence of frames in the captured movie. On the first frame, we initially identify a spatially isolated oligomer or peptide by a Gaussian distributed fluorescence profile from a 9 × 9-pixel matrix. Each pixel was then corrected for the background signal and the 15 brightest pixel values within the matrix were summed to give the oligomer intensity at the specific time at which the movie frame was acquired. This process was then repeated for each oligomer and each time point in the movie. The time series of the summed and background-corrected pixel intensities then produced a fluorescence time trajectory from each oligomer, similar to the trace shown in Fig. 2d. The number of fluorescent dyes (and thus peptides-per-oligomer) present in each fluorescence trajectory may then be determined by counting the number of discrete photobleaching events evident above the background signal.

**Fig. 10:**
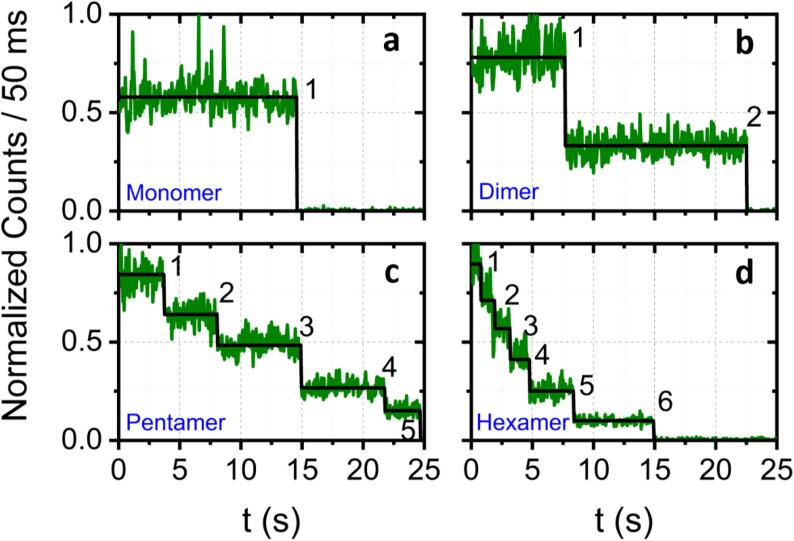
Representative single-molecule stepwise-photobleaching trajectories and reconstructions obtained from immobilized Aβ_555_ oligomers. Normalized variation in the fluorescence emission intensity obtained as a function of time from immobilized Aβ_555_ complexes displaying (a) a single photobleaching event, (b) two photobleaching events, (c) five photobleaching events and (d) six photobleaching events corresponding to the presence of a monomer, dimer, pentamer and hexamer, respectively. Solid green lines correspond to the experimentally measured data and solid black lines correspond to the idealized trajectories obtained via the HMM model.

Baseline-subtracted photobleaching time traces displaying <10 quantized steps were fitted with a hidden Markov model (HMM)[76] to extract the number of peptides-per-oligomer in a probabilistic manner. Recently, HMM has been applied to access discrete levels in protein-induced fluorescence enhancement time traces [77], differentiate between single-molecule FRET states [78], [79], [80] and to analyse multichromophore photobleaching trajectories [81], [82]. Briefly, HMM is a stochastic model that maps measured values to unobserved states. In this application, the fluorescence trajectories obtained from single Aβ_555_ oligomers were modelled as a sequence of up to 10 different intensity values and transitions between these states were defined as a photobleaching event. HMM utilizes the Viterbi algorithm [83] to find the most likely sequence of states given a set of data and corresponding transition probability matrix and emission probability functions. We used the HaMMY algorithm [76] to analyse each normalized Aβ_555_ trajectory. The majority of photobleaching traces displayed exponential kinetics, meaning the final photobleaching step in the trajectory generally lasted longer than previous steps, and thus fitting of the last step had the greatest data support. The method was applied to the processing of all experimental stepwise photobleaching trajectories to extract the true number of states. Representative trajectories and reconstructed HMM fits are shown in Fig. 10. By using the HMM model the number of peptides-per-oligomer were then extracted based on the goodness-of-fit and used to construct 1D population distribution histograms from an entire data set.

### 2.6 Optimization of imaging conditions

It is important to note that wide-field single-molecule fluorescence detection does not discriminate between oligomers non-specifically bound to the substrate or fluorescent impurities attached to the glass or in the solvent. It is therefore important to evaluate the inherent background fluorescence within pre-cleaned and passivated microchannels prior to starting any experiment. Impurities in the cleaning agents or buffers are typically the cause of high background levels and no more than 1–2 adsorbed fluorescent species per 25 × 25 μm field of view should be observed prior to the injection of the sample. If a high number of fluorescent spots are observed, then all buffers and solvents should be imaged to check for fluorescent impurities.

Aβ oligomerization is known to be extremely sensitive to small variations in the environmental conditions [24], [25] and therefore the choice of aggregation buffer conditions are critical to consistently obtain reproducible results. The acceleration of Aβ oligomerization has already been observed at the interface of biomimetic membranes, and lipid rafts, and it has been suggested that when interfaced with highly fluorinated compounds, similar to HFIP, oligomers associated with the appearance of cognitive problems [73] are formed. Importantly the buffer conditions are also chosen to enable oligomer binding to the poly-l-lysine surface, and minimize any unwanted time-dependent changes to the dye photophysics. For these experiments, pH regulation is also essential, as it has been shown that exposure of Aβ to slightly acidic conditions accelerates the formation of higher order species.

### 2.7 Data Interpretation. A case study on the evolution and inhibition of Aβ_555_ oligomers.

**Fig. 11:**
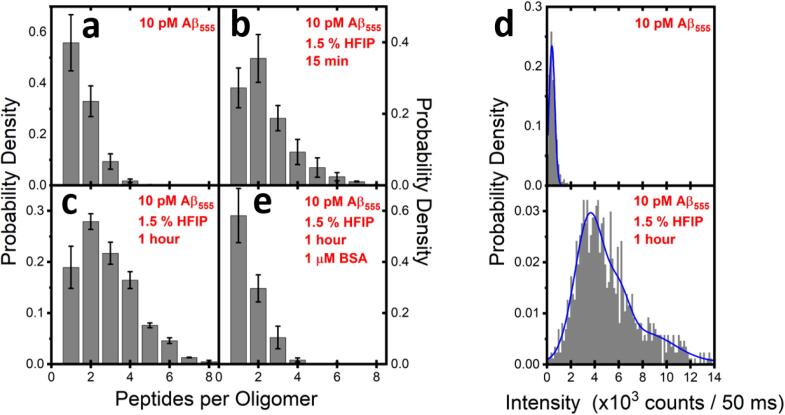
Distributions of Aβ_555_ monomers and oligomers under varying sample conditions, as determined by single-molecule stepwise-photobleaching and intensity analysis. The number of peptides per fluorescent spot as determined by HMM analysis as a function of environmental condition are shown. Distributions were obtained from a freshly prepared sample of 10 pM Aβ_555_ in (a) 50 mM Tris buffer (pH8) and (b) after injection of 1.5% (v/v) HFIP after 15 min and (c) 1 h. (d) Representative Aβ_555_ intensity histograms are shown at the beginning (top panel) and end stage (bottom panel) of the oligomerization process. As expected, the oligomerized Aβ_555_ sample has an intensity distribution shifted towards higher values than for the freshly prepared sample. (e) Also shown are the number of peptides-per-oligomer observed for a 10 pM sample of Aβ_555_ in the presence of 1.5% (v/v) HFIP and the oligomer inhibitor BSA (1 μM) after 1 h incubation. Data shown in (a-c and e) is expressed as the mean ± the standard error of the mean from three experimental runs. The histograms in (a), (b), (c) and (e) were constructed from a total of 6808, 1333, 4308 and 1631 fluorescent spots, respectively.

A sample data set that we obtained from the stepwise photobleaching assay is shown in Fig. 11. In essence, each 1D histogram shows the frequency of oligomeric Aβ_555_ species within a given set of environmental parameters. In the absence of HFIP, a freshly prepared 10 pM Aβ_555_ solution in 50 mM Tris buffer (pH 8.0) deposited directly onto a poly-l-lysine surface displayed probability densities of 0.56 ± 0.11 corresponding to monomeric species (one-step bleaching) and 0.33 ± 0.06 corresponding to the presence of dimers (two-step bleaching). Only a small fraction of trimers and higher-order oligomers were observed by comparison (Fig. 11 a). This distribution is in good agreement with that reported for FAM-labelled Aβ(1–40) in PBS buffer at pH 7.4, and serves to demonstrate that even under conditions considered to be ‘non-aggregating’ (i.e. lacking an aggregation catalyst), a distribution of monomers and low-order oligomers rapidly form and must therefore be properly accounted for in any subsequent analysis. It is important to note that control experiments indicate that surface-bound poly-l-lysine nor base buffer influences the distribution of the species over the course of 5 h after preparation. To investigate the potential of the stepwise photobleaching method to monitor the time-dependent oligomerization of Aβ_555_, we next explored the fluorescence response from the labelled-peptides under experimental conditions known to promote globular oligomers. In this case, the addition of 1.5% (v/v) of HFIP to a freshly-prepared solution of 10 pM Aβ_555_ in 50 mM Tris buffer (pH 8) induced a progressive decrease in the overall number of monomers, and a progressive increase in the number of oligomers over the course of 1 h (Fig. 11 b, c). One of the advantages of the stepwise photobleaching approach is that the growth of Aβ_555_ oligomers can also be identified by changes in the intensity distribution obtained from immobilized peptides and oligomers. For example, Fig. 11 d shows the intensity distribution from individual Aβ_555_ complexes at the beginning and end-point of the oligomerization process. As expected, the intensity distribution is shifted to higher values, indicating an increase in the relative abundance of oligomerized species. The reproducibility of the assay was confirmed by performing three replicates using different Aβ_555_ preparations.

To test the ability of the stepwise photobleaching method to report the interaction between Aβ and inhibitory agents, we investigated the effect of bovine serum albumin (BSA) as a model system. BSA is the most abundant protein found in mammalian serum and while its primary function is to transport various ligands, mounting evidence also supports a role as a molecular chaperone to prevent the misfolding and aggregation of amyloidogenic proteins [84], [85]. With respect to Aβ, BSA has displayed strong inhibition of the fibrillization process [84], [86] and was used in this work to explore its inhibitory potential towards HFIP-induced oligomers. As shown in Fig. 11 e, a 10 pM Aβ_555_ sample incubated with 1 μM BSA was predominantly monomeric even after 1 h in the presence of 1.5% (v/v) HFIP. To get a clearer picture of whether the sample population exhibited significant differences relative to freshly prepared Aβ_555_ in the absence of HFIP and BSA (Fig. 11 a), we performed a two-way ANOVA on the population means with Aβ species and condition as factors. This analysis revealed that at the p = 0.05 level, the population means associated with 10 pM Aβ_555_ are not significantly different to the means observed after 1 h in the presence of 1 μM BSA (F = 0.795, p = 0.438). This is expected taking into account that the relative difference in mean probability densities for the monomeric and dimeric forms are only 0.02 and 0.03, respectively.

Taken together, these results demonstrate that stepwise photobleaching of the covalently attached dye in the monomer and aggregated forms can be easily determined, thereby providing a means to quantify variations in relative populations as a function of time or oligomerization conditions. Such differentiation is not possible using ensemble-based analyses or extrinsic probes that do not interact with monomeric and/or oligomeric forms of Aβ. This is important to note given several lines of evidence that suggest amyloid toxicity may not be directly related to the presence of single oligomers, but rather the ability of these objects to grow and dynamically exchange [87].

### 2.8 Fluorescence assays for assessing cell viability and intracellular Ca^2+^ levels following exposure of cells to Aβ oligomers.

The mechanisms linking Aβ to neuronal dysfunction and eventual cell death are under-explored and remain largely obscure [88]. However, the emergence of recent experimental evidence supporting a toxicity model in which different soluble Aβ oligomers formed at different stages of the self-assembly process induce membrane permeabilization prompted us to apply a ratiometric and intracellular calcium sensing strategy to test for oligomer activity. We hypothesise that oligomer-induced intracellular Ca^2+^ overload (or indeed reduction) could be a no-return signal that triggers neuronal death.

The method utilizes low concentrations of the calcium-sensitive (k_d_ = 0.14 μM) and cell-permeant fluorescent ratiometric indicator, Fura-2, containing an acetoxymethyl ester (AM) group. Cleavage of the AM moiety by cytoplasmic esterases traps the dye within cells. The indicator undergoes spectral shifts following Ca^2+^ binding, enabling free Ca^2+^ levels to be quantified within living cells [88]. Fura-2-AM is one of the most common ratiometric calcium indicators and has an emission peak at 512 nm. When free cytosolic Ca^2+^ binds, the excitation peak shifts from 380 nm to 340 nm, while the peak emission at 512 nm remains unchanged. By sequentially exciting Fura-2-AM at 340 nm and 380 nm, and taking the ratio of the emission intensities at each excitation wavelength, a measurement of the corresponding cytosolic Ca^2+^ concentration can be gauged if the ratio has also been calibrated against known free Ca^2+^ concentrations. Unlike many other ratiometric Ca^2+^ probes, Fura-2-AM was chosen in this work because it has a larger dynamic range and offers greater resistance to photobleaching.

It is worth noting that single cell microscopy imaging is one setting in which Fura-2-AM has been extensively applied to enable the simultaneous measurement of Ca^2+^ response in numerous individual cells incubated with Aβ. In this regard, the treatment of Fura-2-AM loaded astrocytes with Aβ(25–35) fragments and serotonin has been shown to increase Ca^2+^ signaling [89], and recently Aβ(1–42) oligomers were found to aggravate the loss of store operated Ca^2+^ entry and increase the resting cytosolic Ca^2+^ concentration in aging neurons [90]. Increased Aβ(1–42) activity has also been measured in elevated glucose levels as detected via Fura-2-AM loaded rat neurons [91]. Moreover, Fura-2 derivatives loaded into large unilamellar vesicles have been used to suggest a two-step mechanism of Aβ(1–40) induced membrane disruption, whereby initial pore formation precedes membrane fragmentation [92], [93]. However, although the presence of oligomeric species were typically reported via, for example, Western Blots, heterogeneity between oligomeric forms were not fully characterized. Having established a platform from which to access heterogeneity within oligomeric populations via stepwise photobleaching in this work, we next measured the ratiometric fluorescence response of neuroblastoma cells loaded with Fura-2-AM in the presence of pre-characterized Aβ oligomers, as shown schematically in Fig. 13.

**Fig. 12:**
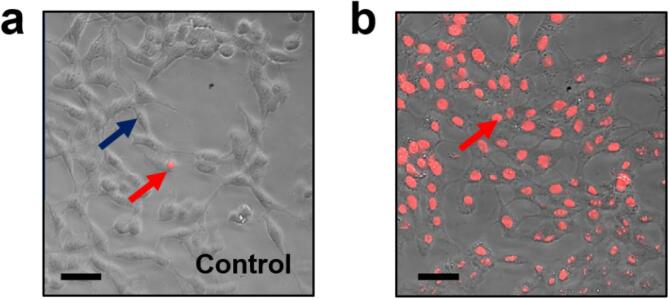
Propidium iodide staining in B104 cells. Nuclear morphological changes on treatment with (a) the absence and (b) presence of 2.5 μg/mL Aβ(1–42) under oligomerization conditions (KREBS buffer, 0.5% HFIP, 1% DMSO) for 2 h in B104 neuroblastoma cells were detected by propidium iodide staining and observed via fluorescence microscopy. The blue arrow indicates a live cell, whereas red arrows represent apoptotic cells with altered nuclei (shown in red). The scale bar indicates 5 μm.

To assay the activity of freshly prepared oligomers, the following procedure should be adopted:

1. Human or rodent neuroblastoma cells (we use the rat B104 cell line [94]) are maintained as adherent cultures in Dulbecco’s modified eagle medium (DMEM) containing glutamine supplementation (Thermo Fisher Scientific, UK, part number 11965084), 10% (v/v) foetal bovine serum (FBS) and 100 U/ml penicillin/streptomycin (Thermo Fisher Scientific, UK, part number 15140122) in sterile 75 cm^2^ flasks (Corning, Germany, part number 430639) at 37 °C, 5% CO_2_. The cells are passaged when confluent, usually every 2–3 days, by transferring a third of the cells to a new flask.
2. The fluorescence imaging assays are conducted in a 96 well plate. To plate cells, they must first be detached from the flask and counted. Remove the DMEM media from the flask by pipette and gently wash the cells by replacing the media with 10 ml phosphate-buffered saline (PBS; 0.137 mM NaCl, 2.7 mM KCl, 10 mM Na_2_HPO_4_, 1.8 mM KH_2_PO_4_, pH 7.4).
  Remove the PBS buffer by pipette and add 1 ml 0.025% trypsin and 0.01% EDTA in PBS buffer to the flask (Thermo Fisher Scientific, UK, part number R001100). Return the flask to the incubator for 5 min. This step detaches the cells from the flask surface. After a maximum of 5 min, add 10 ml DMEM to the flask and remove the cell suspension to a 15 ml centrifuge tube. Pellet the cells by spinning at 1000 g for 5 min in a centrifuge.
3. Remove the supernatant and resuspend the cell pellet in 1 ml of DMEM. To evaluate the cell density, apply a 20 μL sample from the cell suspension to a haemocytometer (VWR, UK, part number 631–0696). View the cells using a conventional phase contrast brightfield upright microscope (eg. Motic, UK, AE20 series) at room temperature. Count the number of cells in a 0.1 × 0.1 mm^2^ area (equivalent to a volume of 0.1 mm^3^) and multiply by 10, 000 to obtain the number of cells / ml.
4. Dilute the cells in DMEM to yield a concentration of 25, 000 / ml and apply 200 μL (5000 cells) to each well in a 96 well-plate (Corning, Germany, part number 3988). Place the plate in the incubator overnight.
5. Prior to treating the cells, they are equilibrated in the assay buffer. Remove the culture media from each well and replace with 200 µL KREBS buffer (119 mM NaCl, 2.5 mM KCl, 1 mM NaH_2_PO_4_, 2.5 mM CaCl_2_·2H_2_O, 1 mM MgCl_2_·6H_2_O, 4 mM HEPES, 10 mM D-glucose, pH 7.4). Incubate the cells for 10 min at 37 °C.
6. To assay the effect of Aβ oligomers on cell viability, cells were treated for 2 h at 37 °C in KREBS buffer containing 4–10 μg/mL of Aβ(1–42) (unlabelled) or Aβ_555_ (labelled) in < 0.5% (v/v) HFIP and < 1% (v/v) DMSO, with 0.1 μM propidium iodide (PI) (Sigma Aldrich, UK, part number P4170). PI is a nuclear and chromosome fluorescent stain with excitation and emission maxima at 535 nm and 617 nm, respectively, when bound to DNA. PI is not membrane permeable in live cells, but intercalates between DNA bases with little dependence on sequence when membrane permeabilization occurs. The cells in the 96 well plate are imaged every few minutes over the 2 h incubation period. Imaging is conducted with an epi-fluorescence microscope (eg. Nikon, Japan, part number TE200) using a 20 X objective (eg. Nikon, part number ELWD 20XO), 550 nm excitation filter (Chroma, USA, part number ET550/15x), 575 nm dichroic mirror (Nikon, Japan, part number G2-A DM575) and a 590 nm long pass emission filter (Nikon, Japan, G2-A, BAA590). Images are captured in brightfield and fluorescence modes using a CCD camera (eg. Rolera-XR, Q Imaging, Canada) with a 200 ms exposure. Brightfield and fluorescence images can be combined using the ‘Merge’ function in ImageJ and cell viability determined by counting the number of dead cells displaying co-localized regions of fluorescence occurring from PI stained nuclei. After 2 h incubation with 4–10 μg/mL of Aβ(1–42) (unlabelled) or Aβ_555_ (labelled), cell viability progressively dropped to < 20% (Fig. 12), pointing towards a membrane permeabilization mode of action. Under control conditions, in which B104 cells were treated with KREBS buffer including 0.5% (v/v) HFIP and 1% (v/v) DMSO but lacking Aβ(1–42), no change to cell viability was observed over the course of 2 h (Fig. 12).

**Fig. 13:**
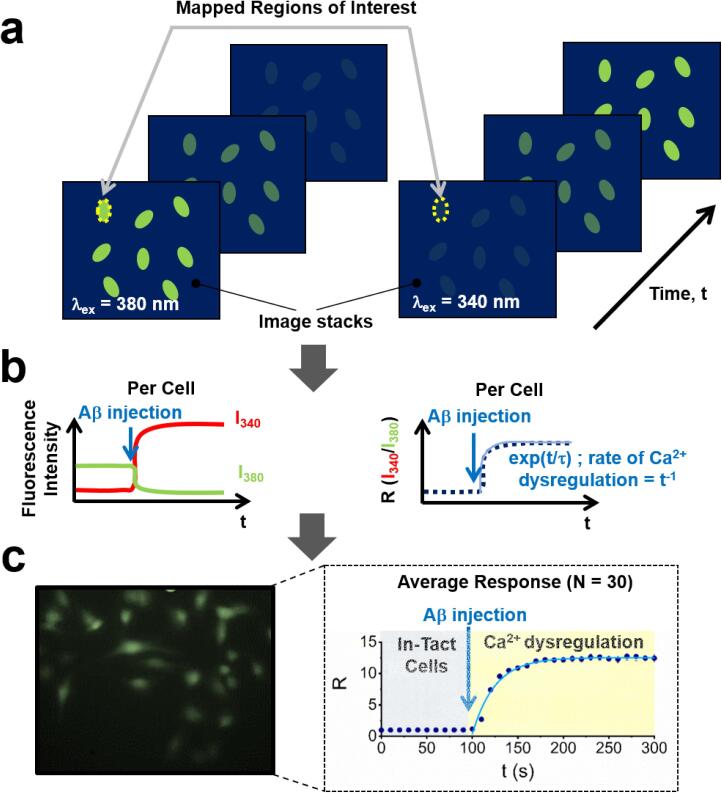
The process of data collection and analysis for determining Aβ-induced Ca^2+^ dysregulation in B104-neuroblastoma cells. (a) Wide-field fluorescence images of Fura-2-AM loaded cells obtained with excitation wavelengths of 380 nm and 340 nm are taken over time and recorded as image stacks. In each movie frame, corresponding pairs of regions of interest are identified. (b) Each pair in each movie frame gives the fluorescence intensities, I_380_ and I_340_, associated with Fura-2-AM emission. A time-trajectory of the fluorescence emission intensities is used to generate the Ratio of emission intensities, R, as a function of time. (c) Representative wide-field fluorescence image of Fura-2-AM loaded B104 neuroblastoma cells (λ_ex_ = 340 nm) at 200 s after application of 2.5 μg/ mL pre-formed Aβ_555_ oligomers. Detailed descriptions can be found in the main text. Also shown is the mean R response from 30 cells after Aβ application. Error bars represent the standard error of the mean.
7. To assay the dysregulation of intracellular Ca^2+^ by Aβ oligomers, cells are first incubated with 0.1 μM Fura-2-AM (Thermo Fisher Scientific, UK, part number F1201) and 0.001% pluronic acid in KREBS buffer for 20 min at 37 °C.
8. After 20 min incubation, gently wash each well three times with 200 µL KREBS buffer at room temperature to remove any residual dye. After the third wash step, incubate the cells in KREBS buffer for 10 min at 37 °C prior to treatment with Aβ oligomers and live cell imaging.
9. Imaging was performed using the same microscope as specified in Step 6, with 340 and 380 nm excitation filters (Chroma, USA, part numbers ET340x and ET380x), a 400 nm dichroic mirror (Chroma, USA, part number T400lp) and 510 nm band pass emission filter (Chroma, USA, part number ET510/80 m) replaced. Images are acquired in brightfield and fluorescence modes, with the latter performed sequentially using excitation wavelengths of 340 nm and 380 nm, respectively. Fura-2-AM fluorescence emission is collected at 510 nm, using an exposure time of 200 ms. Imaging is typically performed every 10 s over a time-window of 1000 s after application of Aβ-containing KREBS buffer.
10. Image stacks containing fluorescence from Fura-2-AM under excitation of 340 nm and 380 nm can be produced using the ‘Stack’ function in ImageJ (Fig. 13a). Cells that take up Fura-2-AM are identified by comparing brightfield images with fluorescence images, and subsequently defined as regions of interest. Image stacks are then imported into Fiji and fluorescence trajectories per cell are determined using the ‘Time Series Analyzer’ plugin.
11. The dysregulation of calcium homeostasis induced by Aβ is observed as an exponential increase in the ratio of the fluorescence emission intensity using 340 nm excitation and a corresponding decrease in fluorescence intensity 380 nm excitation (Fig. 13b). Intracellular calcium dysregulation is typically observed immediately after application of pre-formed HFIP-Aβ_555_ oligomers onto Fura-2-AM loaded cells, and followed via a single-exponential growth model with a kinetic rate constant of 0.028 ± 0.003 s^−1^ (χ^2^ = 0.97) (Fig. 13c). We note that a limitation to Fura-2-AM, as with most Ca^2+^ indicators, is that it cannot be specifically targeted to intracellular organelles. However, recent advances in probe designs have seen the development of mitochondrial-specific derivatives of Fura-2 [95], and these may be valuable given the strong body of evidence linking Aβ with mitochondrial dysfunction [96]. Nevertheless, the absolute concentration of free calcium per cell, C, at each time point, can be related to the dissociation constant via the expression

$$C=k_{d}\frac{\left(R-R_{\min}\right)}{\left(R_{\max}-R\right)}\frac{I_{f}}{I_{b}}$$

where R is the ratio of the fluorescence emission obtained under 340 and 380 nm excitation, respectively. The values of R_max_ and R_min_ are the ratio values measured under conditions of saturating calcium levels and the absence of calcium, respectively. I_f_ and I_b_ are the fluorescence emission intensities of free and bound Fura-2 obtained using 380 nm excitation.

In essence, this procedure assays a sequence of events whereby Aβ -induced membrane permeabilization promotes an imbalance in intracellular Ca^2+^ levels preceding neuronal death. Since dysregulation in Ca^2+^ homeostasis is observed long before the onset of the first symptoms of AD, this relatively straightforward protocol may offer possibilities for evaluating the rate of Ca^2+^ dysregulation as a function of oligomeric morphology, and may even be extended to evaluating the effects on peripheral cells such as fibroblasts and lymphocytes, offering a far greater understanding of the role of Aβ oligomers in AD pathogenesis. A proper understanding of the complexity of Ca^2+^ signalling clearly requires careful evaluation of the applied scientific approach, and the knowledge acquired from this protocol, does not allow one to categorically state whether alterations in Ca^2+^ homeostasis are the key and primary event in AD, or whether they simply accompany the AD pathway. It should also be noted that this protocol is discussed in the context of understanding Aβ toxicity under controlled conditions and this may be indistinct from the pathology observed in the brain of an AD patient. Nevertheless, it is clear that instabilities in Ca^2+^ homeostasis appear early in AD pathogenesis, and the presented strategy may at least have some relevance to the disease process, and we expect the approach could ultimately aid basic science methods aimed at correcting or slowing Aβ-induced dysregulation of Ca^2+^ homeostasis.

## 3 Conclusions and outlook

As evident from our results and others, single-molecule stepwise photobleaching measurements can provide unprecedented levels of detail and insight into the stoichiometry of single immobilized Aβ oligomers, providing important insight in particular when correlated with ratiometric information which details oligomer activity. By utilizing Aβ(1–42) labelled at the N-terminal position with a fluorophore, we have demonstrated that the population distributions of oligomers, as well as their time-dependent evolution, can be easily accessed, overcoming several limitations associated with traditional sensing methods involving extrinsic probes. Importantly, the technique not only constitutes a powerful and straightforward approach to quantify the size distribution of single oligomers in solution, but also provides a quantifiable metric to ensure that samples of starting Aβ material are identical. This is a crucial feature to avoid common discrepancies observed with *in vitro* studies of Aβ aggregation caused by heterogeneity between freshly prepared samples [97]. While the use of intrinsic probes are not completely free from interference limitations, they offer interesting advantages over the use of extrinsic probes. Since in this case HiLyte Fluor 555 is covalently linked to the Aβ monomer at the N-terminus, the identification of oligomeric species is not limited by the formation of probe-specific binding sites or by any variations in the affinity of the extrinsic probe. Moreover, the use of an intrinsic probe potentially eliminates competition between extrinsic probes and inhibitor compounds for binding sites, however, as with any fluorescence-based assay, care must be taken to ensure that any alterations of the fluorescence properties of the intrinsic probe are not due to specific interactions with the inhibitor. There are of course obvious limitations to the use of N-terminal labelling for certain applications. For instance, the requirement to employ synthetic peptides makes difficult its implementation as a diagnostic tool, and while there is evidence to suggest that N-terminal tagging with HiLyte Fluor 555 and others does not perturb aggregation rates or the final surface-immobilized aggregated structures [24], [98], [99], it is important to note that we cannot rule out that the immobilized Aβ activity is different from the native form. Nevertheless, experimental tools capable of accessing oligomer stoichiometry are critical for a full understanding of the environmental factors which govern their growth and stability, and although the stepwise photobleaching method at the current time is only applicable to controlled experimental conditions devoid of contaminants and solutions with complex composition, we expect it may be useful for the rational screening of novel anti-oligomerization inhibitors *in vitro*. Moreover, by evaluating the kinetic rate of Aβ -induced dysregulation of intracellular Ca^2+^ homeostasis, we have demonstrated a ratiometric approach to quantify the oligomeric activity. Taken together, the approaches presented enable direct characterization of the earliest stages of Aβ(1–42) self-assembly and toxicity to be accessed, bypassing major limitations associated with conventional sensing strategies. More generally, the implementation of correlative single-molecule based techniques represents a complementary approach for the real-time interrogation of Aβ self-assembly and toxicity mechanisms and is an attractive alternative to the use of extrinsic probes when rationally screening for oligomerization inhibitors. We expect the methods presented here will facilitate investigating the mechanisms of Aβ oligomerization under controlled conditions, and consequently how the growth and toxicity of transient species may be regulated by local environmental variables such as pH and temperature. We also expect the methodology to be extended to the investigation of aggregation processes associated with other misfolded peptides implicated in various pathologies, including tau in Alzheimer’s disease and α-syncuclein in Parkinson’s disease.

## CRediT authorship contribution statement

**Lara Dresser:** Investigation, Formal analysis. **Patrick Hunter:** Investigation, Formal analysis. **Fatima Yendybayeva:** Investigation, Formal analysis. **Alex L. Hargreaves:** Methodology, Writing - original draft, Writing - review & editing. **Jamieson A.L. Howard:** Investigation, Writing - review & editing. **Gareth J.O. Evans:** Data curation, Resources, Formal analysis, Writing - review & editing. **Mark C. Leake:** Data curation, Resources, Formal analysis, Writing - review & editing. **Steven D. Quinn:** Conceptualization, Methodology, Investigation, Formal analysis, Writing - original draft.
